# Comorbid health conditions and their impact on social isolation, loneliness, quality of life, and well-being in people with dementia: longitudinal findings from the IDEAL programme

**DOI:** 10.1186/s12877-023-04601-x

**Published:** 2024-01-05

**Authors:** Serena Sabatini, Anthony Martyr, Anna Hunt, Laura D. Gamble, Fiona E. Matthews, Jeanette M. Thom, Roy W. Jones, Louise Allan, Martin Knapp, Christina Victor, Claire Pentecost, Jennifer M. Rusted, Robin G. Morris, Linda Clare

**Affiliations:** 1 Institute of Mental Health, School of Medicine, University of Nottingham, Nottingham, UK; 2 University of Exeter Medical School, University of Exeter, Exeter, UK; 3 Population Health Sciences Institute, Newcastle University, Newcastle, UK; 4 Faculty of Medicine and Health, The University of Sydney, Sydney, Australia; 5 Research Institute for the Care of Older People (RICE), Bath, UK; 6 London School of Economics and Political Science, London, UK; 7 College of Health, Medicine and Life Sciences, Department of Health Sciences, Brunel University London, London, UK; 8 School of Psychology, University of Sussex, Brighton, UK; 9 King’s College Institute of Psychiatry, Psychology and Neuroscience, London, UK; 10 NIHR Applied Research Collaboration South-West Peninsula, Exeter, UK

**Keywords:** Physical health, Comorbidity, Illnesses, Social network, Alzheimer’s disease

## Abstract

**Background:**

Most people with dementia have multiple health conditions. This study explores (1) number and type of health condition(s) in people with dementia overall and in relation to age, sex, dementia type, and cognition; (2) change in number of health conditions over two years; and (3) whether over time the number of health conditions at baseline is related to social isolation, loneliness, quality of life, and/or well-being.

**Methods:**

Longitudinal data from the IDEAL (Improving the experience of Dementia and Enhancing Active Life) cohort were used. Participants comprised people with dementia (*n* = 1490) living in the community (at baseline) in Great Britain. Health conditions using the Charlson Comorbidity Index, cognition, social isolation, loneliness, quality of life, and well-being were assessed over two years. Mixed effects modelling was used.

**Results:**

On average participants had 1.8 health conditions at baseline, excluding dementia; increasing to 2.5 conditions over two years. Those with vascular dementia or mixed (Alzheimer’s and vascular) dementia had more health conditions than those with Alzheimer’s disease. People aged ≥ 80 had more health conditions than those aged < 65 years. At baseline having more health conditions was associated with increased loneliness, poorer quality of life, and poorer well-being, but was either minimally or not associated with cognition, sex, and social isolation. Number of health conditions had either minimal or no influence on these variables over time.

**Conclusions:**

People with dementia in IDEAL generally had multiple health conditions and those with more health conditions were lonelier, had poorer quality of life, and poorer well-being.

**Supplementary Information:**

The online version contains supplementary material available at 10.1186/s12877-023-04601-x.

## Background

An estimated 55 million people worldwide are living with dementia [1]. Dementia involves a significant and progressive decline in one or more cognitive domains, such as memory and/or language, and concomitant difficulties in everyday activities [2]. Studies conducted in Europe and in North America show that approximately 90% of people with a diagnosis of dementia have at least one additional comorbid condition, and the number of comorbid health conditions increases as people age [3–10]. A recent systematic review and meta-analyses found that just over half of the global population aged over 60 have at least one health condition [11]. Importantly, a scoping review, comprising mostly US and UK studies, found that people with dementia with comorbid health conditions have less access to treatment and monitoring for non-dementia health conditions (e.g., diabetes) than people with similar comorbidities but without dementia [4]. The authors hypothesize this may be due to problems attending appointments due to memory difficulties. It may also be that when people with dementia present with co-occurring behavioral symptoms, as these can be more distressing managing these may be a greater priority for consultants compared to treating health conditions that are more physical [12, 13]. However, some behavioral issues might co-occur with health problems such as pain or sleep disturbance thus treating underling health conditions may be necessary to resolve co-occurring behavioral issues [14, 15].

Lack of detection and/or treatment of comorbid health conditions in people with dementia leads to numerous negative outcomes for people with dementia, caregivers, and the health care system [16]. Indeed, people with dementia with more comorbid health conditions are at risk of faster decline in cognitive and functional abilities, greater mortality, poorer quality of life (QoL) and well-being [4, 17–20]. Lack of management and/or treatment of comorbid health conditions in people with dementia also increases caregiver burden [21]. Moreover, as people with more health conditions use more health care services [3, 22] and are more frequently hospitalized [23–25], lack of management and/or treatment of comorbid health conditions in people with dementia increases health service costs [26, 27]. Early detection, management, and treatment of comorbid health conditions in people with dementia is therefore a high priority. As longitudinal evidence on comorbidity in people with dementia is scarce [4, 5, 28], investigating changes in the number and type of comorbid health conditions in community-dwelling people with dementia is needed in order to gain a better understanding of their health care needs as dementia severity increases [29].

The negative impact that a higher number of health conditions has on the QoL and well-being of people with dementia at cross-sectional level may exacerbate over time, though studies that explore the impact of health conditions on QoL and well-being over time are scarce [17, 18]. Having comorbid conditions may also increase the social isolation and loneliness of people with dementia, and these may have a further negative impact on the QoL and well-being of people with dementia. Indeed, the physical limitations associated with certain health conditions may make social interactions more difficult which may lead to a concomitant and progressive decrease in social interactions over time. While social isolation indicates objective lack of social relationships and interactions, loneliness concerns the discrepancy between one’s actual relationships and the expected/desired quantity and quality of relationships [30, 31]. Although social isolation and loneliness do not always overlap, loneliness appears more common among people with dementia who are socially isolated [32].

This study aims to (1) describe the number and type of health conditions over two-years in a large sample of people with dementia living in Great Britain and in relation to their age, sex, dementia type, and severity of cognitive impairment, (2) test whether number of health conditions changes over two years, and (3) investigate the associations between the number of health conditions at baseline with social isolation, loneliness, QoL, and well-being over time.

## Methods

### Study design

This study used baseline (2014-16); 12-month (2015-17) and 24-month (2016-18) follow-up data from the Improving the experience of Dementia and Enhancing Active Life (IDEAL) programme [33]. Analyses were conducted using version 7 of the datasets. People with dementia and their caregivers, where available, were recruited through a network of 29 National Health Service (NHS) sites in England, Scotland, and Wales. Participants of any age could enroll in IDEAL if they lived in the community, had a diagnosis of any type of dementia, and had a Mini-Mental State Examination [34] score ≥ 15 at baseline, indicating mild-to-moderate dementia. Exclusion criteria were people who at baseline had a comorbid terminal illness and/or were unable to provide informed consent. At any timepoint, participants were excluded where there was potential for home visits to pose risk to research network staff. Further details about IDEAL are reported in the study protocol [33]. The IDEAL study was approved by the Wales 5 Research Ethics Committee (reference: 13/WA/0405) and the Ethics Committee of the School of Psychology, Bangor University (reference: 2014-11684), and is registered with the UK Clinical Research Network (registration number: 16593).

### Instruments

All measures were administered at all timepoints and self-rated by people with dementia except where indicated.

*Health conditions* were assessed with the Charlson Comorbidity Index (CCI) [35, 36]. For people with dementia with a caregiver taking part in the study, the CCI was administered jointly to people with dementia and their caregivers at baseline and was completed by caregivers at 12-month and 24-month follow-up. When the person with dementia did not have a caregiver in the study, the CCI was self-completed by the person with dementia. Health conditions were included in the CCI based on their link with mortality [36]. The CCI was expanded in 2008 by the inclusion of four additional conditions [35]. Example health conditions included in the CCI are cerebrovascular disease, dementia, diabetes, and cancer within the last five years and if present whether the cancer had metastasized. Some conditions are considered as superordinate and contain some subtypes; for instance, cerebrovascular disease is treated as superordinate with stroke, cerebrovascular accident, and transient ischemic attack as specific subordinate conditions. A count of the 22 superordinate conditions reported, excluding dementia but including leukemia and lymphoma, was used as an indicator of the number of comorbid health conditions (possible score: 0-22) [7].

*Personal characteristics* comprised age, sex, marital status, living situation, and dementia type.

*Marital status* comprised four categories: single (never married); married (including people who married for a second time or who were in a civil partnership); divorced (including those who were separated); widowed (never remarried).

*Living situation* comprised four categories: living alone; living with spouse/partner; living with others [37]; living in residential care. Living in residential care was applicable only at 12-months and 24-months as living in residential care was an exclusion criterion at study entry.

*Dementia type* comprised seven subcategories formally diagnosed by a clinician: Alzheimer’s disease; vascular dementia; mixed Alzheimer’s disease and vascular dementia; frontotemporal dementia; Parkinson’s disease dementia; dementia with Lewy bodies; unspecified/other [38]. Diagnostic information was recorded by research staff from medical records.

*Cognition* was assessed with the Mini-Mental State Examination [34]. Lower scores (possible range: 0-30) indicate more severe cognitive impairment [39].

*Social network* was assessed with the 6-item Lubben Social Network Scale [31]. Total scores (possible range 0-30) were dichotomized into “socially isolated” (0-11) and “not socially isolated” (12-30) [31].

*Loneliness* was assessed with the 6-item De Jong Gierveld & Tilburg scale [30]. Higher scores (possible range: 0-6) indicate greater loneliness. This measure was administered at baseline and 24-month follow-up only.

*Quality of life* was assessed with the 13-item Quality of Life in Alzheimer’s Disease scale [40]. This is a widely-used measure of quality of life in people with dementia [17], and enquires about aspects of their current situation such as memory. Higher scores (possible range: 13-52) indicate greater QoL.

*Well-being* was assessed with the World Health Organization-Five Well-Being Index [41]. This is a widely-used measure of well-being [42], that investigates aspects of psychological well-being. Higher scores (possible range: 0-100) indicate greater well-being.

### Analyses

Descriptive statistics for study variables and for each health condition listed in the CCI at baseline, 12-month, and 24-month follow-up were reported.

A nested case-control approach was used to determine whether number of health conditions was associated with dropout. This involved logistic regression models to determine whether dropout was associated with number of health conditions at the previous timepoint. Estimates were combined using *metan* in Stata [43].

Mixed effects modelling with a Poisson distribution was used to investigate the trajectory of change in number of health conditions over two years, and to explore differences at baseline and over the two years across subgroups based on age, sex, dementia type, and severity of cognitive impairment. The models estimated rate ratios (RR) for the intercept and a slope, with random effects to account for variation across individuals, and were adjusted for age, sex, and dementia type. Linear mixed effects modelling was used to examine the associations of number of health conditions at baseline with QoL and well-being at baseline and over two years. Generalized linear mixed models were used to examine the associations of number of health conditions at baseline with social isolation (binomial distribution) and loneliness (gamma distribution). An unadjusted and an adjusted model (age, sex, and dementia type) was fitted for each outcome.

Analyses were conducted using Stata version 17 [44]. Missing data on outcome measures was handled using full-information maximum likelihood estimation.

## Results

### Participant characteristics

Out of the 1545 people with dementia that took part in IDEAL at any of the three timepoints, 55 were excluded from this study as they (and/or their caregivers) did not complete the CCI questionnaire at any timepoint. Hence the present study includes 1490 participants at baseline, 1176 at 12-month follow-up, and 850 at 24-month follow-up. In IDEAL the main reasons for participants’ withdrawal were lack of interest in continuing, poor health of the person with dementia or the carer, and loss to follow-up. At baseline, the mean age of participants was 76.3 years, just over half had Alzheimer’s disease, and just over half were male. Most participants were married and living with a spouse or partner. Sample characteristics at follow-ups were similar to baseline (see Table 1).

**Table 1:** Descriptive statistics for study variables at baseline and at 12-month and 24-month follow-ups

	Baseline (*N* = 1490)	12-month follow-up (*N* = 1176)	24-month follow-up (*N* = 850)
	Statistics		
**Demographic variables**			
Age in years, M (SD)	76.31 (8.49)	77.14 (8.38)	77.52 (8.45)
Age group, n (%)			
< 65 years	131 (8.8)	89 (7.6)	66 (7.8)
65-69	174 (11.7)	128 (10.9)	72 (8.5)
70-74	254 (17.1)	193 (16.4)	159 (18.7)
75-79	355 (23.7)	268 (22.7)	172 (20.2)
≥ 80	576 (38.7)	498 (42.4)	381 (44.8)
Ethnicity, n (%)			
White British	1403 (95.7)	1103 (95.7)	801 (96.2)
White other	46 (3.1)	39 (3.4)	25 (3.0)
Other	17 (1.2)	10 (0.9)	7 (0.8)
Missing	24	24	17
Sex, n (%)			
Women	649 (43.6)	665 (56.6)	375 (44.1)
Men	841 (56.4)	511 (43.4)	475 (55.9)
Marital status, n (%)			
Single	24 (1.6)	15 (1.3)	13 (1.5)
Married/Civil partnership/Cohabiting	1120 (75.2)	891 (75.8)	643 (75.7)
Divorced/Separated	91 (6.1)	75 (6.4)	57 (6.7)
Widowed	255 (17.1)	195 (16.5)	137 (16.1)
Living situation, n (%)			
Living alone	279 (18.7)	199 (16.9)	134 (15.8)
Live with spouse/partner	1127 (75.7)	887 (75.7)	641 (75.5)
Live with other	82 (5.6)	66 (5.6)	45 (5.3)
Living in residential care	0 (0)	23 (2.0)	29 (3.4)
Missing/unclassifiable, n	2	1	1
Dementia type, n (%)			
Alzheimer’s disease	832 (55.8)	658 (56.0)	488 (57.4)
Vascular dementia	161 (10.8)	115 (9.8)	82 (9.7)
Mixed (Alzheimer’s and vascular)	313 (21.0)	263 (22.4)	185 (21.7)
Frontotemporal dementia	53 (3.6)	39 (3.3)	31 (3.7)
Parkinson’s disease dementia	42 (2.8)	33 (2.8)	17 (2.0)
Dementia with Lewy bodies	50 (3.4)	39 (3.3)	27 (3.2)
Unspecified/other dementia	39 (2.6)	29 (2.4)	20 (2.3)
Mini-Mental State Examination, M (SD)	23.21 (3.60)	21.60 (5.05)	20.53 (6.21)
Missing	1	11	12
Number of health conditions (excluding dementia), M (SD)	1.80 (1.63)	2.23 (1.80)	2.47 (1.87)
Zero health conditions, n (%)	334 (22.7)	173 (15.3)	101 (12.5)
One health condition, n (%)	421 (28.6)	272 (24.1)	167 (20.7)
Two health conditions, n (%)	313 (21.3)	277 (24.5)	204 (25.3)
Three health conditions, n (%)	203 (13.8)	180 (15.9)	132 (16.5)
Four health conditions, n (%)	101 (6.9)	106 (9.4)	89 (11.1)
Five health conditions, n (%)	55 (3.7)	54 (4.8)	57 (7.1)
Six health conditions, n (%)	20 (1.4)	31 (2.7)	26 (3.2)
Seven health conditions, n (%)	15 (1.0)	23 (2.1)	14 (1.7)
Eight health conditions, n (%)	6 (0.4)	8 (0.7)	9 (1.1)
Nine health conditions, n (%)	4 (0.2)	6 (0.5)	4 (0.5)
Ten health conditions, n (%)	0 (0)	0 (0)	3 (0.4)
Missing, n	18	46	44
Social network, n (%)			
Socially isolated	412 (28.6)	328 (29.8)	239 (32.0)
Not socially isolated	1030 (71.4)	773 (70.2)	509 (68.0)
Missing, n	48	75	102
Loneliness, M (SD)^a^	1.34 (1.48)		1.42 (1.49)
Missing, n	89		88
Quality of life, M (SD)	36.84 (5.93)	36.99 (5.87)	36.94 (5.63)
Missing, n	156	140	136
Well-being, M (SD)	61.01 (20.52)	60.92 (20.65)	61.28 (20.96)
Missing, n	23	55	70

^a^Loneliness was assessed only at baseline and the 24-month follow-up timepoint

### Number and type of health conditions over two years

Mean number of health conditions, excluding dementia, was 1.8 at baseline. The mixed effects model adjusted for age, sex, and dementia type showed an increase in number of health conditions over two years (RR = 1.17; 95% CI: 1.14, 1.21), leading to an average of 2.5 health conditions at 24-month follow-up.

At baseline, in addition to dementia, 77.7% of participants had at least one CCI health condition. This proportion increased to 85.3% and 88.2% at 12-month and 24-months follow-up, respectively. At baseline and 12-month follow-up the most health conditions people had were nine, and this increased to ten at the 24-month follow-up. At baseline, the most frequent health conditions were hypertension, present in more than one-third of participants, and inflammation affecting the joints, present in almost one-third of participants. Health conditions that were present for 10% to 15% of participants were depression, diabetes (controlled by insulin or equivalent), chronic bad chest, and cerebrovascular disease. See Table 2 for the details of each health condition.

**Table 2:** Number and percentage of participants having each health condition at baseline, 12-month, and 24-month follow-ups

	Baseline (*N* = 1472)	12-month follow-up (*N* = 1130)	24-month follow-up (*N* = 806)
**Health conditions**			
	Yes, N (%)		
Myocardial infarction (history of heart attacks)	130 (8.8)	128 (11.3)	96 (11.9)
Congestive heart failure	67 (4.6)	68 (6.0)	53 (6.6)
Hypertension/high blood pressure	566 (38.5)	509 (45.0)	390 (48.4)
Diagnosed depression	221 (15.0)	212 (18.8)	187 (23.2)
Peripheral vascular disease	152 (10.3)	183 (16.2)	157 (19.5)
Aortic aneurysm	19 (1.3)	20 (1.77)	18 (2.2)
Poor circulation	93 (6.3)	116 (20.3)	107 (13.3)
Cerebrovascular disease	228 (15.5)	216 (19.1)	166 (20.6)
Stroke	110 (7.5)	105 (9.3)	79 (9.8)
Cerebrovascular accident	7 (0.5)	7 (0.6)	4 (0.5)
Transient Ischemic attack	108 (7.3)	124 (11.0)	104 (12.9)
Chronic bad chest	200 (13.6)	181 (16.0)	134 (16.6)
Asthma	103 (7.0)	99 (8.8)	74 (9.2)
Chronic obstructive pulmonary disease	53 (3.6)	54 (4.8)	38 (4.7)
Chronic bronchitis	19 (1.3)	20 (1.8)	19 (2.4)
Emphysema	14 (1.0)	19 (1.7)	16 (2.0)
Inflammation affecting the joints	434 (29.5)	416 (36.8)	340 (42.2)
Lupus	2 (0.1)	2 (0.2)	0 (0)
Rheumatoid arthritis	205 (13.9)	233 (20.6)	212 (26.3)
Connective tissue disease	10 (0.7)	10 (0.9)	5 (0.6)
Vasculitis	8 (0.5)	11 (1.0)	8 (1.0)
Peptic/stomach ulcer disease	59 (4.01)	54 (0.4)	42 (5.2)
Skin ulcer	24 (1.6)	35 (3.1)	31 (3.8)
Bed sores	4 (0.3)	6 (0.5)	3 (0.4)
Repeated cellulitis	8 (0.5)	13 (1.2)	13 (1.6)
Diabetes controlled with insulin or equivalent	189 (12.8)	171 (15.1)	132 (16.4)
Diabetes with end organ damage	29 (2.0)	23 (2.0)	29 (3.6)
Damage to the retina	12 (0.8)	13 (1.2)	11 (1.4)
Nerve damage	6 (0.4)	10 (0.9)	12 (1.5)
Kidney damage	9 (0.6)	7 (0.6)	9 (1.1)
Brittle diabetes	2 (0.1)	2 (0.2)	2 (0.2)
Moderate or severe chronic kidney disease	38 (2.6)	39 (3.5)	34 (3.0)
Hemiplegia	3 (0.2)	5 (0.4)	4 (0.5)
Cancer within the last five years	134 (9.1)	134 (11.9)	115 (14.3)
Breast cancer	28 (1.9)	24 (2.1)	18 (2.2)
Colon cancer	11 (0.7)	16 (1.4)	15 (1.9)
Prostate cancer	45 (3.1)	44 (3.9)	29 (3.6)
Lung cancer	4 (0.3)	3 (0.3)	4 (0.5)
Skin cancer	19 (1.3)	20 (1.8)	23 (2.9)
Blood cancer/lymphoma	4 (0.3)	5 (0.4)	6 (0.7)
Acute or chronic leukemia	0 (0)	1 (0.1)	2 (0.2)
Cancer within the past five years that has metastasized	7 (0.5)	12 (1.1)	12 (1.5)
Mild liver disease	5 (0.3)	7 (0.6)	5 (0.6)
Hepatitis B	0 (0)	5 (0.4)	0 (0)
Hepatitis C	0 (0)	0 (0)	0 (0)
Cirrhosis	2 (0.1)	2 (0.2)	1 (0.1)
Liver disease (moderate to severe)	1 (0.1)	3 (0.3)	0 (0)
Chronic jaundice	1 (0.1)	1 (0.1)	0 (0)
Liver failure	1 (0.1)	2 (0.2)	0 (0)
Liver transplant	0 (0)	0 (0)	0 (0)
AIDS or HIV	0 (0)	0 (0)	0 (0)
Taking warfarin	154 (10.5)	115 (10.2)	74 (9.2)

In the present study we were interested in the count of health conditions. However, conditions that are typically weighted as having a higher impact on health and the cost of care are hemiplegia, moderate or severe chronic kidney disease, diabetes with end of organ damage, any cancer, skin ulcers/cellulitis, moderate or severe live disease, metastatic cancer, and AIDS or HIV. In the weighted CCI total score all these conditions receive a weight of two except for moderate or severe liver disease which receives a weight of three and metastatic cancer and AIDS or HIV which receive a weight of six. The remaining conditions, and taking warfarin, receive a weight of one [35]

### Number of health conditions according to age, sex, dementia type, and severity of cognitive impairment

There was no association between number of health conditions and sex (Table 3). Those aged ≥ 80 were more likely to have more health conditions than those < 65 years at baseline. Compared to people with Alzheimer’s disease, those with vascular dementia or mixed Alzheimer’s disease and vascular dementia were more likely to have more health conditions at baseline; see Supplementary Table 1 for the number and proportion of participants having each of the investigated health conditions for each dementia type. There was little evidence of an association between cognition and number of health conditions. There was no association between age, sex, dementia type, or cognition with change in number of health conditions over time.

**Table 3:** Association of age, sex, dementia types, and cognitive impairment with number of health conditions

	Number of health conditions	
	Mean intercept (rate ratio, 95% CI)	Mean slope (rate ratio, 95% CI)
Sex (reference: male)		
Female	0.91 (0.84, 1.00)	1.00 (0.94, 1.06)
Age group (reference: < 65 years)		
65-69	1.07 (0.89, 1.30)	0.96 (0.82, 1.12)
70-74	1.07 (0.90, 1.30)	0.98 (0.85, 1.13)
75-79	1.17 (0.98, 1.40)	0.98 (0.85, 1.13)
≥ 80	1.25 (1.05, 1.48)	0.99 (0.87, 1.12)
Dementia type (reference: Alzheimer’s disease)		
Vascular dementia	1.79 (1.55, 2.03)	0.98 (0.89, 1.07)
Mixed (Alzheimer’s and vascular)	1.49 (1.34, 1.67)	0.99 (0.92, 1.07)
Frontotemporal dementia	0.09 (0.70, 1.16)	1.11 (0.91, 1.34)
Parkinson’s disease dementia	1.08 (0.84, 1.40)	0.99 (0.79, 1.23)
Dementia with Lewy bodies	1.02 (0.80, 1.30)	0.99 (0.82, 1.21)
Unspecified/other dementia	1.00 (0.76, 1.32)	0.96 (0.77, 1.21)
Cognition (Mini-Mental State Examination)	1.01 (1.00, 1.03)	1.00 (0.99, 1.01)

A nested case-control approach was used to determine whether number of health conditions was associated with dropout from the study. This analysis suggests that number of health conditions was not associated with dropout (combined estimate odds ratio, OR = 0.99; 95% CI: 0.94, 1.05; T1-T2 OR = 0.99; 95% CI: 0.91, 1.07; and T2-T3 OR = 1.00; 95% CI: 0.93, 1.07).

### Associations of number of health conditions with social isolation, loneliness, quality of life, and well-being over two years

The proportion of people with dementia who were socially isolated increased over the two years (adjusted model OR = 1.26; 95% CI: 1.00, 1.58). Loneliness (adjusted model RR = 1.01; 95% CI: 0.98, 1.05), QoL (adjusted model mean slope = -0.33; 95% CI: -0.60, -0.05), and well-being (adjusted model mean slope = -0.61; 95% CI: -1.22, 0.02) did not change significantly over time. How health conditions affected these four variables longitudinally is presented in Table 4. Having a higher number of health conditions at baseline was not associated with social isolation at baseline nor over time. A higher number of health conditions at baseline was associated with greater loneliness at baseline but not over time. At baseline a higher number of health conditions was associated with poorer QoL and poorer well-being. Whilst there was some evidence of small associations for number of health conditions with QoL and well-being over time, Supplementary Figures 1a-b suggest this was regression to the mean, i.e. scores reduce closer to the mean for participants that previously scored higher and scores increase closer to the mean for participants that previously scored lower.

**Table 4:** Association of number of health conditions with social isolation, loneliness, quality of life and wellbeing

	Social network Odds ratio (95% CI)	Loneliness Rate ratio (95% CI)	Quality of life estimate (95% CI)	Well-being estimate (95% CI)
Unadjusted model				
Intercept (CCI baseline)	1.06 (0.97, 1.17)	1.03 (1.01, 1.04)	-0.71 (-0.85, -0.57)	-2.33 (-2.82, -1.84)
Slope (CCI x time)	0.96 (0.88, 1.04)	1.00 (0.99, 1.01)	0.19 (0.09, 0.28)	0.45 (0.09, 0.81)
Adjusted model				
Intercept (CCI baseline)	1.03 (0.93, 1.14)	1.03 (1.01, 1.04)	-0.71; (-0.85, -0.56)	-2.37 (-2.86, -1.88)
Slope (CCI x time)	0.96 (0.89, 1.04)	1.00 (0.99, 1.01)	0.18; (0.08, 0.28)	0.46 (0.10, 0.81)

*CCI* Charlson Comorbidity Index

Adjusted for age, sex, and dementia type

## Discussion

This study explored the number and type of CCI health conditions in people with dementia living in Great Britain in relation to age, sex, dementia type, and cognitive difficulties. This study also investigated whether the number of CCI health conditions changed over two years and whether over the same time period number of CCI health conditions was related to social isolation, loneliness, QoL, and/or well-being. On average people with dementia had approximately two health conditions at baseline, excluding dementia, and this number increased by on average half an additional health condition over two years. Similarly, to the general population of older adults in England [45], the most frequent conditions were hypertension, inflammation affecting the joints, and diagnosed depression. Only those aged ≥ 80 were more likely to have a higher number of health conditions than those < 65, indicating that the number of comorbid conditions in people with dementia significantly increases after the age of 80, consistent with previous research [8]. Compared to people with Alzheimer’s disease, those with vascular dementia or mixed Alzheimer’s disease and vascular dementia were more likely to have a higher number of health conditions at baseline. At baseline, those with more cognitive difficulties were slightly less likely to have more health conditions than those with fewer cognitive difficulties, even after controlling for age. However, the effect size was very small and may have been due to sample characteristics as, among those with more cognitive difficulties, only those with fewer health conditions may have been willing to take part in the study. Change in number of health conditions over time did not differ among subgroups based on age, sex, dementia type, or cognition. A higher number of health conditions at baseline was not related to social isolation, loneliness, QoL, and/or well-being over two years.

A population-based study estimated that in the UK 91% of people with dementia have at least one additional comorbid condition [3]. This proportion is higher than that found in the present study where the proportion of participants having at least one health condition, excluding dementia, was 77.7% at baseline, though interestingly the proportion was more similar at 12-month follow-up (85.3%), and at 24-month follow-up (88.2%). The higher proportion reported in the earlier study [3] may be due to their participants being on average 5 years older than those included in the present study. Indeed, as people in the present study aged the proportion increased and people with dementia aged 80 and over reported the highest number of health conditions. The slightly lower proportion of comorbid health conditions found in the present study may also be due to methodological differences; the present study considered health conditions reported by people with dementia and their caregivers included in the CCI whereas the earlier study [3] used data from clinical registers, thus data were not restricted to 22 conditions as in the present study. Nonetheless, the high proportions of people with dementia with at least one comorbid health condition in the present study and in the earlier study [3] demonstrates the importance of accounting for co-occurring conditions in people with dementia when planning personalized care [46, 47], especially those conditions considered severe enough to be included in the CCI, which was primarily devised to measure increased risk of mortality.

At baseline people with vascular dementia or mixed (Alzheimer’s and vascular) dementia had more health conditions than other dementia types. However, people with vascular dementia or mixed (Alzheimer’s and vascular) dementia were not at greater risk of increase in the number of health conditions over time than those with other dementia types. It may be that vascular neuropathology is associated with specific needs [48]. Indeed, compared to the other dementia types, in the present study the proportions of people with vascular dementia or mixed (Alzheimer’s and vascular) dementia having hypertension, peripheral vascular disease, and diabetes controlled with insulin were higher. This highlights the importance of tailored healthcare and management strategies for people with different dementia types, taking into account their unique medical profiles and needs.

Findings from the present study suggest that people with dementia with more health conditions at baseline did not have an increase in social isolation or loneliness, nor a decline in QoL or well-being over two years, than people with dementia with fewer baseline health conditions. Nonetheless, people with dementia already had approximately two comorbid health conditions at baseline and those with more health conditions had poorer QoL and poorer well-being at baseline [7, 49], suggesting that greater comorbidity may assert a negative influence on the QoL and well-being of people with dementia. These findings emphasize the importance of addressing comorbid health conditions in people with dementia to improve their QoL and well-being. The findings also highlight the need for continued research into the complex relationships between health conditions, social isolation, and other factors that influence living with dementia.

A limitation of the present study is that health conditions were not retrieved from medical records; it is possible that participants over- or under-reported the number of comorbid health conditions [50]. Moreover, other than depression, psychiatric/mental health conditions were not investigated as these are not included in the CCI. Another limitation is that the present study focused on number of conditions rather than type/severity of conditions. However, few people with dementia had CCI conditions that are weighted for having a higher health impact. Indeed, 0.1% and 0.5% of participants reported having moderate or severe liver disease or metastatic cancer, respectively. Additionally, studies with longer follow-up periods are needed to better delineate how comorbidity changes as dementia severity increases, since people in the present study remained for the most part in the mild-to-moderate stages of dementia. Longer study durations would enable a more thorough examination of the specific health conditions that may progressively exacerbate in people with dementia, particularly those aged 80 or above. By investigating the potential link between dementia and increased comorbidity, we could gain a more comprehensive understanding of the disease progression and develop improved management strategies. Nonetheless, a strength of the study is the large sample of people with dementia, the different dementia types that are often not included in comorbidity research studies, and that the study investigated, for the first time, whether number of health conditions affect social isolation, loneliness, QoL, and well-being over time.

## Conclusions

Generally, people with mild-to-moderate dementia reported approximately two CCI health conditions at baseline, and this number increased by just under one additional health condition over two years. People with dementia aged 80 or over had the most comorbid health conditions. The study also found that having more health conditions was related to poorer QoL and well-being, but only minimally to greater loneliness. These findings highlight the importance of considering multimorbidity in people with dementia, especially those aged 80 or more, so that people with dementia can receive appropriate ameliorative treatment and better maintain good QoL and well-being. Healthcare providers and policy makers need to be aware of the increased risk of comorbid health conditions in people with dementia. It is important to assess the overall health of people with dementia to facilitate the management of multimorbidity and to ensure appropriate personalized treatment and care.

### Supplementary Information

**Additional file 1: Supplementary Table 1.** Number and percentage of participants having each health condition at baseline according to dementia type. **Supplementary Figure 1.** Visualization of the mean intercept and slopes of a) quality of life and b) well-being by number of health conditions.

## Data Availability

IDEAL data were deposited with the UK Data Archive in April 2020. Details of how to access the data can be found here: https://reshare.ukdataservice.ac.uk/854293/.

## Abbreviations

QoL: Quality of Life
IDEAL: Improving the experience of Dementia and Enhancing Active Life
CCI: Charlson Comorbidity Index
OR: Odds ratio
RR: Rate ratio
95% CI: 95% Confidence interval
