# Supplementary material for: Comorbid health conditions and their impact on social isolation, loneliness, quality of life, and well-being in people with dementia: longitudinal findings from the IDEAL programme
Source: BMC Geriatr. 2024 Jan 5;24:23. doi: 10.1186/s12877-023-04601-x (PMC10768096; doi:10.1186/s12877-023-04601-x)

**Supplementary Table 1.** Number and percentage of participants having each health condition at baseline according to dementia type.

| **Health conditions** | Alzheimer’s disease  (N=832) | Vascular dementia  (N=161) | Mixed (Alzheimer’s and vascular)  (N=313) | Frontotemporal dementia  (N=53) | Parkinson’s disease dementia  (N=42) | Dementia with Lewy bodies  (N=50) | Unspecified/other dementia  (N=39) |
| --- | --- | --- | --- | --- | --- | --- | --- |
|  | Yes, N (%) | | |  |  |  |  |
| Myocardial infarction (history of heart attacks) | 54 (6.5) | 27 (16.8) | 34 (10.9) | 3 (5.7) | 3 (7.1) | 2 (4) | 7 (17.9) |
| Congestive heart failure | 21 (2.5) | 14 (8.7) | 21 (6.7) | 2 (3.8) | 2 (4.8) | 1 (2) | 6 (15.4) |
| Hypertension/high blood pressure | 273 (32.8) | 95 (59.0) | 136 (43.5) | 16 (30.2) | 9 (21.4) | 15 (30) | 22 (56.4) |
| Diagnosed depression | 115 (13.8) | 34 (21.1) | 36 (11.5) | 5 (9.4) | 12 (28.6) | 5 (10) | 14 (35.9) |
| Peripheral vascular disease | 62 (7.5) | 27 (16.8) | 46 (14.7) | 4 (7.5) | 3 (7.1) | 1 (2) | 9 (23.1) |
| Aortic aneurysm | 8 (1.0) | 1 (0.6) | 9 (2.9) | 0 (0) | 0 (0) | 0 (0) | 1 (2.6) |
| Poor circulation | 40 (4.8) | 14 (8.7) | 27 (8.6) | 4 (7.5) | 1 (2.4) | 1 (2) | 6 (15.4) |
| Cerebrovascular disease | 55 (6.5) | 60 (37.3) | 84 (26.8) | 3 (5.7) | 5 (11.9) | 7 (14) | 14 (35.9) |
| Stroke | 24 (2.9) | 39 (24.2) | 35 (11.2) | 2 (3.8) | 1 (2.4) | 2 (4) | 7 (17.9) |
| Cerebrovascular accident | 3 (0.4) | 1 (0.6) | 3 (1.0) | 0 (0) | 0 (0) | 0 (0) | 0 (0) |
| Transient Ischemic attack | 28 (3.4) | 25 (15.5) | 37 (11.8) | 1 (1.9) | 3 (7.1) | 5 (10) | 9 (23.1) |
| Chronic bad chest | 101 (12.1) | 24 (14.9) | 48 (15.3) | 7 (13.2) | 3 (7.1) | 8 (16) | 9 (23.1) |
| Asthma | 57 (6.9) | 9 (5.6) | 21 (6.7) | 3 (5.7) | 2 (4.8) | 7 (14) | 4 (10.3) |
| Chronic obstructive pulmonary disease | 17 (2.0) | 7 (4.3) | 22 (7.0) | 2 (3.8) | 1 (2.4) | 2 (4) | 2 (5.1) |
| Chronic bronchitis | 9 (1.1) | 3 (1.9) | 3 (1.0) | 1 (1.9) | 0 (0) | 0 (0) | 3 (7.7) |
| Emphysema | 12 (1.4) | 0 (0) | 1 (0.3) | 0 (0) | 0 (0) | 1 (2) | 0 (0) |
| Inflammation affecting the joints | 233 (28.0) | 49 (30.4) | 97 (31.0) | 10 (18.9) | 10 (23.8) | 18 (36) | 17 (43.6) |
| Lupus | 1 (0.1) | 0 (0) | 0 (0) | 0 (0) | 0 (0) | 1 (2) | 0 (0) |
| Rheumatoid arthritis | 108 (13.0) | 22 (13.7) | 46 (14.7) | 3 (5.7) | 8 (19.0) | 9 (18) | 9 (23.1) |
| Connective tissue disease | 4 (0.5) | 1 (0.6) | 3 (1.0) | 1 (1.9) | 0 (0) | 1 (2) | 0 (0) |
| Vasculitis | 5 (0.6) | 0 (0) | 3 (1.0) | 0 (0) | 0 (0) | 0 (0) | 0 (0) |
| Peptic/stomach ulcer disease | 29 (3.59) | 8 (5.0) | 15 (4.8) | 3 (5.7) | 1 (2.4) | 0 (0) | 3 (7.7) |
| Skin ulcer | 11 (1.3) | 1 (0.6) | 9 (2.9) | 0 (0) | 1 (2.4) | 1 (2) | 1 (2.6) |
| Bed sores | 1 (0.1) | 1 (0.6) | 1 (0.3) | 0 (0) | 1 (2.4) | 0 (0) | 0 (0) |
| Repeated cellulitis | 3 (0.4) | 0 (0) | 3 (1.0) | 0 (0) | 1 (2.4) | 1 (2) | 0 (0) |
| Diabetes controlled with insulin or equivalent | 83 (10.0) | 27 (16.8) | 63 (20.1) | 2 (3.8) | 1 (2.4) | 6 (12) | 7 (17.9) |
| Diabetes with end organ damage | 12 (1.4) | 7 (4.3) | 8 (2.6) | 0 (0) | 1 (2.4) | 0 (0) | 1 (2.6) |
| Damage to the retina | 4 (0.5) | 3 (1.9) | 4 (1.3) | 0 (0) | 0 (0) | 0 (0) | 1 (2.6) |
| Nerve damage | 2 (0.2) | 3 (1.9) | 1 (0.3) | 0 (0) | 0 (0) | 0 (0) | 0 (0) |
| Kidney damage | 5 (0.6) | 2 (1.2) | 2 (0.6) | 0 (0) | 0 (0) | 0 (0) | 0 (0) |
| Brittle diabetes | 2 (0.2) | 0 (0) | 0 (0) | 0 (0) | 0 (0) | 0 (0) | 0 (0) |
| Moderate or severe chronic kidney disease | 17 (2.0) | 6 (3.7) | 12 (3.8) | 0 (0) | 1 (2.4) | 0 (0) | 2 (5.1) |
| Hemiplegia | 0 (0) | 0 (0) | 1 (0.3) | 0 (0) | 1 (2.4) | 0 (0) | 1 (2.6) |
| Cancer within the last five years | 84 (10.1) | 11 (6.8) | 28 (8.9) | 1 (1.9) | 5 (11.9) | 2 (4) | 3 (7.7) |
| Breast cancer | 21 (2.5) | 1 (0.6) | 3 (1.0) | 0 (0) | 2 (4.8) | 0 (0) | 1 (2.6) |
| Colon cancer | 6 (0.7) | 2 (1.2) | 2 (0.6) | 0 (0) | 0 (0) | 1 (2) | 0 (0) |
| Prostate cancer | 25 (3.0) | 5 (3.1) | 12 (3.8) | 0 (0) | 2 (4.8) | 1 (2) | 0 (0) |
| Lung cancer | 2 (0.2) | 0 (0) | 1 (0.3) | 0 (0) | 0 (0) | 0 (0) | 1 (2.6) |
| Skin cancer | 11 (1.3) | 1 (0.6) | 3 (1.0) | 0 (0) | 0 (0) | 0 (0) | 4 (10.3) |
| Blood cancer/lymphoma | 3 (0.4) | 0 (0) | 1 (0.3) | 0 (0) | 0 (0) | 0 (0) | 0 (0) |
| Acute or chronic leukemia | 0 (0) | 0 (0) | 0 (0) | 0 (0) | 0 (0) | 0 (0) | 0 (0) |
| Cancer within the past five years that has metastasized | 3 (0.4) | 0 (0) | 2 (0.6) | 1 (1.9) | 0 (0) | 0 (0) | 1 (2.6) |
| Mild liver disease | 3 (0.4) | 0 (0) | 2 (0.6) | 0 (0) | 0 (0) | 0 (0) | 0 (0) |
| Hepatitis B | 0 (0) | 0 (0) | 0 (0) | 0 (0) | 0 (0) | 0 (0) | 0 (0) |
| Hepatitis C | 0 (0) | 0 (0) | 0 (0) | 0 (0) | 0 (0) | 0 (0) | 0 (0) |
| Cirrhosis | 1 (0.1) | 0 (0) | 1 (0.3) | 0 (0) | 0 (0) | 0 (0) | 0 (0) |
| Liver disease (moderate to severe) | 0 (0) | 0 (0) | 1 (0.3) | 0 (0) | 0 (0) | 0 (0) | 0 (0) |
| Chronic jaundice | 0 (0) | 0 (0) | 1 (0.3) | 0 (0) | 0 (0) | 0 (0) | 0 (0) |
| Liver failure | 0 (0) | 0 (0) | 1 (0.3) | 0 (0) | 0 (0) | 0 (0) | 0 (0) |
| Liver transplant | 0 (0) | 0 (0) | 0 (0) | 0 (0) | 0 (0) | 0 (0) | 0 (0) |
| AIDS or HIV | 0 (0) | 0 (0) | 0 (0) | 0 (0) | 0 (0) | 0 (0) | 0 (0) |
| Taking warfarin | 65 (7.8) | 19 (11.8) | 47 (15.0) | 2 (3.8) | 7 (16.7) | 6 (12) | 8 (20.5) |

**Supplementary Figure 1.** Visualization of the mean intercept and slopes of a) quality of life and b) well-being by number of health conditions.

A B


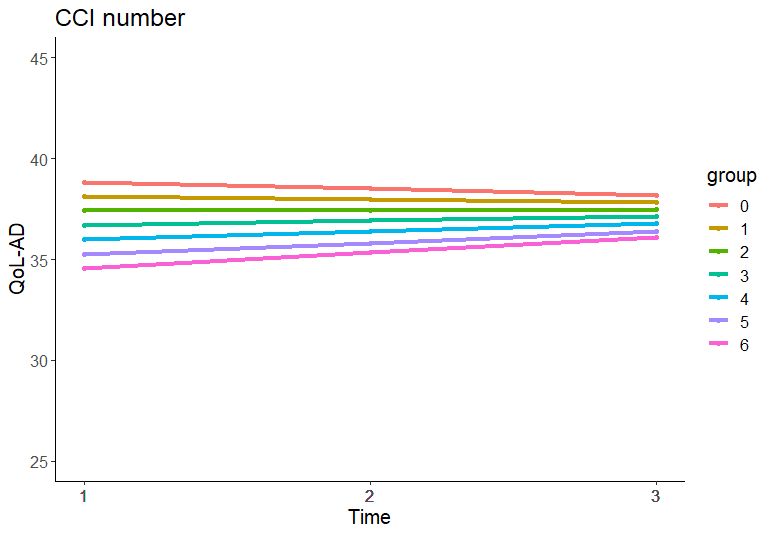

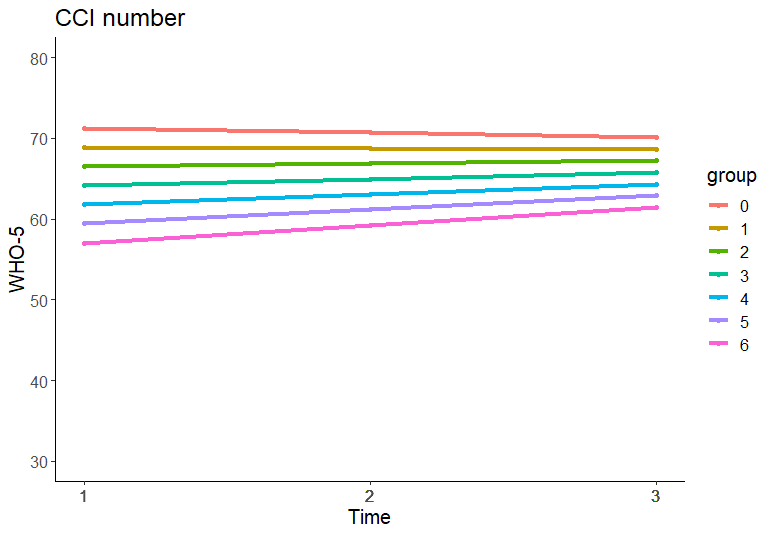

Supplement: Supplementary file 1 — Additional file 1: Supplementary Table 1. Number and percentage of participants having each health condition at baseline according to dementia type. Supplementary Figure 1. Visualization of the mean intercept and slopes of a) quality of life and b) well-being by number of health conditions. [file 12877_2023_4601_MOESM1_ESM.docx]
